# Focus on Liability of Residences for Elderly and Sick People: A Case Series and Medico-Legal Issues

**DOI:** 10.3390/healthcare11040539

**Published:** 2023-02-11

**Authors:** Tommaso D’Anna, Antonina Argo, Giuseppe Davide Albano, Maria Puntarello, Chiara Rizzo, Daniela Guadagnino, Stefania Zerbo

**Affiliations:** 1 Policlinic Hospital, University of Palermo, Via del Vespro 129, 90127 Palermo, Italy; 2 Department of Health Promotion, Mother and Child Care, Internal Medicine and Medical Specialties, Section of Legal Medicine, University of Palermo, 90127 Palermo, Italy

**Keywords:** elderly people residence, healthcare liability, missed care, disability, social–health management, medico-legal issues, guidelines, medical liability

## Abstract

Residences for elderly and sick people, self-sufficient or dependent, are varied. To date, the liability profiles of these structures are not clearly delineated, and increasingly often, their operating and organization criteria are entrusted to subnational, regional, or local regulations. Among the various deficits, there is the keeping of a complete and detailed documentation/diary of the patient, the lack of which can generate medico-legal problems. In this paper, we present three cases of guests in residences for a dependent person brought to the attention of the Institute of Forensic Medicine of the University Hospital of Palermo due to criminal proceedings, where the lack of existing documentation in the structure and, in some cases, the behavior of the professionals working there, led the evaluator to deduce the organization’s culpability.

## 1. Introduction

There are various types of residences for elderly and sick people, which differ from each other in various characteristics. Specifically, long-term care facilities are organizations dedicated to dependent elderly people who need medical, nursing, and rehabilitation assistance on an ongoing basis. A long-term care facility, on the other hand, houses elderly people who are partially self-sufficient but affected by acute pathologies that require continuous assistance from specialized personnel. Finally, a rest home is used to accommodate self-sufficient or partially self-sufficient elderly people, as underlined in some Italian standards, such as the Decree of the President of the Sicilian Region of 29 June 1988 [1]. As described in this decree, the aim is to guarantee a collective and social life, as well as basic healthcare, for all guests of the organization; the purpose of the establishment of rest homes does not differ much, even internationally, as it is focused more on the social support of the guest rather than on the health of the guest [2]. However, the organization of the aforementioned structures varies in different countries, such as in the USA, in relation to economic–health management policy. The international scientific literature underlines the need to improve the quality of life of older people in need of care and assistance, appropriate management of frailty, and respect for older people’s fundamental rights and freedoms [3,4,5]. Indeed, according to European guidelines, the adequacy and high-standard assistance of older people in long-term care is a prerogative of the health system and is a relevant public health issue [3,4,5].

In Italian legislation, such as the Ministerial Decree of the Minister for Social Solidarity no. 308 of 21 May 2001 [6], reference is mainly made to the requirements that a rest home must have to be defined as such, but little attention is focused on the obligations that the facility must have towards its guests, about the type of healthcare that should be provided, and how this should be documented. Moreover, apart from the basic legislation, each organization has its own internal regulations that can differ, complicating the search for a single procedure to follow.

Clearly, the lack of specific legislation undermines the jurisprudential system that has to judge behaviors and/or activities placed into practice by employees of semi-residential organizations in the absence of clear guidelines. However, useful considerations for implementing the social–health management of residents of residential/rest homes are provided by data from the international scientific literature, and these data underline the need for clearer and more extensive guidelines referring to the global assistance of the host to make their interpretation unambiguous and thus improve patient management and reduce the cases of liability of fragile patients hosted in such organizations. The evaluation of medical liability in the case of patients residing in long-term care facilities is based on adherence to clinical guidelines and good clinical practices of care [7,8,9]. This paper aims to emphasize the medico-legal issues of the management of elderly people in long-term care facilities and the relevant topics of medical malpractice liability in such cases by presenting three cases that came to the attention of the Institute of Forensic Medicine of the University of Palermo in the context of penal criminal processes activity for the Prosecutor Office.

## 2. Case Series

### 2.1. Case 1

A 91-year-old woman suffering from multiple infarct brain syndrome with cerebral atrophy and cognitive decline, arterial hypertension, cervical and knee arthrosis, lumbarspondylolisthesis, and severe osteoporosis was admitted to a nursing home following the worsening of her clinical condition with the onset of aggression and psychomotor agitation. Once the patient was stabilized, she was then transferred to a rest home that was supposed to fully manage the needs of the patient.

Although the patient was not self-sufficient, according to Italian legislation, it is possible to accept non-self-sufficient guests to the extent of 20% of the total number of beds in the facility. During the guest’s stay in the facility, the health services provided to the lady did not appear suitable, and this was also true of the hygiene services, as reported in a complaint filed by the patient’s relatives. In fact, in the documentation, it was clear that the nurse who worked at the rest home had decided not to treat one of the bedsores in the sacral region anymore, as it was too deep, and instead waited for management to pass the case on the integrated home care (ADI) services provided by the public health system. The activation of ADI takes several days, the passage of which undoubtedly led to a worsening of the patient’s clinical conditions to the point of causing her total disability, with the need to place a bladder catheter which then became permanent. The sore appeared infected with the bacterium *Proteus Mirabilis*. Even though the organization of the residence was then changed and all the required health and social–health practices were implemented, the status of the guest by then had reached a point that did not allow her significant clinical improvement other than the stabilization of the pressure ulcers and the resolution of the infection.

In this regard, while no culpability was identified for the other health professionals who treated the guest in the rest home, this was different for the nurse who arbitrarily decided not to treat the pressure ulcer, as his conduct was reprehensible and negligent.

### 2.2. Case 2

A 91-year-old woman, chronically bedridden, suffering from Type II diabetes mellitus, arterial hypertension, hypertensive heart disease, dysphagia, and severe obesity, following the onset of general malaise and a transient loss of consciousness, alerted the on-call doctor at the continuity center for local assistance. The doctor diagnosed a hypertensive crisis which was treated pharmacologically. Despite therapy, there was no improvement in the symptoms; therefore, her family members decided to take her to the emergency room of the nearby hospital. At admission, the suspected diagnosis of ictus cerebri was confirmed. During her hospitalization, she was treated appropriately and was discharged with a diagnosis of right fronto-temporo-parietal ischemic stroke of an atherothrombotic nature, with residual left facio-brachio-crural hemiplegia, motor and sensorineural aphasia, and a nasogastric tube in site, with therapy to be carried out at home and rehabilitation through ADI. Upon her discharge, her family decided to transfer her to a nursing home.

In the residential facility, the patient should have received the treatment prescribed on discharge and undergone rehabilitation; however, there is no evidence of the existence of a clinical diary or any register of all healthcare acts (e.g., administration of a drug at one time rather than another; provision of nursing assistance, such as the dressing of a wound, the change of a bladder catheter, or the management of a nasogastric tube) useful for the management and clinical course of the lady within of the residence.

The patient’s death occurred 11 days after admission to the rest home. The culpability of the structure was recognized since it had not produced any health documentation certifying the correct management of the guest.

### 2.3. Case 3

A 49-year-old man, suffering from a motor deficit and aphasia following cerebral hemorrhagic lesions from road trauma, was entrusted to the care of a nursing home for non-self-sufficient/disabled people. During his stay at this facility, he experienced a worsening neurological picture with the onset of cerebral ischemia, leaving left hemiplegia associated with urinary incontinence (probably from a neurogenic bladder).

A few years later, during the COVID-19 pandemic, the patient was hospitalized for the onset of fever and dyspnea. The molecular swab for SARS-CoV-2 was positive. On admission, he presented a picture compatible with sepsis. During the hospital stay, diagnostic–therapeutic procedure of the case was carried out. However, after 5 days of hospitalization, he died.

During the abdominal autopsy, signs of peritonitis were found, and after an incision in the right colon, about 20 cm from the ileocecal valve, a dental prosthesis was found relating to the elements of the missing upper left dental arch. The macroscopic and histopathological investigations made it possible to trace the cause of death, which occurred due to rapidly evolving septic shock secondary to intestinal perforation with bacterial peritonitis with a culture of *Klebsiella pneumoniae* and paralytic/mechanical ileus secondary to the presence of a relative dental prosthesis for the missing elements of the left upper dental hemiarch (elements 2.2, 2.3, and 2.4) in a SARS-CoV-2 positive subject.

It is probable that the intestinal perforation secondary to the ingestion of the dental prosthesis occurred before admission to the hospital, i.e., during the stay in the community, given that the subject at the emergency room access presented significant neutrophilic leukocytosis associated with a rise in temperature and inflammation indices and vague gastrointestinal symptoms. Regarding liability profiles, it is believed that the subject in question should have received greater surveillance to avoid such events. Furthermore, an incomplete medical record did not trace the moment of the worsening of the symptoms. Therefore, in the light of these critical issues, a censurable conduct against the community was recognized for not noticing the ingestion of the dental prosthesis.

## 3. Discussion

The description of the three cases sheds light on the health responsibility of long-term care facilities toward guests/patients.

Demographics describe an aging society nationally and internationally. An increasing number of older adults live in long-term care facilities (LTCFs). In 2018, the World Health Organization predicted that approximately 1% of the population in most developing countries would suffer from a disability. For example, according to the Malaysian Department of Statistics 2018, the elderly population is projected to reach approximately 5.1 million by 2030 (Malaysia Department of Statistics 2018). Therefore, nursing homes should be prepared well in advance to meet the needs and improve the criticalities of this population category [10].

According to Italian legislation, a “contractual liability” arises between the structure (debtor) and the guest (creditor), given that the aspiring guest or next of kin signs a contract with the structure classified as a rest home. Being a contractual responsibility, the burden of proof rests with the structure, which in this case, to have greater self-defense, should keep not only registers with the personal details of the guests but also registers from which it is possible to view the therapy to be administered, the times of administration, and at least the global trend of the guest throughout the day, guaranteeing continuous assistance and a correct passage of information among the health operators of the organization itself to support better management of the host as a whole. These activities have greater relevance when the rest home organization assumes the responsibility of hosting fragile, non-self-sufficient patients who need continuous medical and nursing assistance, as in the second case illustrated in this paper. This demonstrates that directives relating to every guest of a specific residential or semi-residential organization should be more stringent. However, little space should be left for the free interpretation of the legislation valid in this field. Moreover, the new legislation regarding the medical liability in Italy (Law “Gelli-Bianco”) deals with malpractice litigation by distinguishing the healthcare worker’s (HCW’s) liability from the liabilities of the healthcare facility itself. Healthcare facility litigation is encouraged rather than direct action against HCWs. HCW practice is evaluated according to adherence to guidelines and good clinical practices recommendations [7,8,9]. The lack of adequate documentation and low medical records quality are significant issues that arise from the presented cases. In two cases, the HCWs could not demonstrate that they had met their duty, given the lack of clinical documentation. Medical records documentation is essential in inpatient medical care. Medical records quality and evidence-based medicine are strictly connected with patient outcomes. Indeed, the quality of clinical documentation reflects a more organized HS. A well-documented written guidance on patient movement and accurate bed management is associated with a lower rate of nosocomial infections. Conversely, inaccurate clinical documentation may contribute to patient safety impairment and higher malpractice risk exposure [7,8,9]. In this regard, medical records quality should be implemented and promoted in all healthcare settings. Guidelines that refer not only to logistical/architectural aspects but, above all, to the management of health in force within the organization (clinical registers, therapy sheets) are necessary both for the protection of the guest and the organization, which should protect itself in the event of malpractice claims. In this regard, a German study by Thederan and colleagues [11] that involved an analysis of the prevalence of the visual impairment, as well as its management, showed that many of these conditions were not treated correctly, and improvement was requested in terms of ophthalmological assistance as well as the personnel who took care of the patients, underlining the fact that there are still health shortages. Furthermore, a comparative study on the influence of occupational dental hygiene on the oral and general health of nursing home residents in September 2021 in the USA (152 participants in two nursing homes) to evaluate the influence of a quarterly treatment of occupational dental hygiene on general and oral health concluded that daily oral hygiene procedures are necessary for the oral health of resident patients in LTCFs [12]. This study effectively underlined the need for frequent interventions to optimize the health policy framework necessary to allow caregivers more time for oral hygiene and to establish the accessibility of frequent professional healthcare for the inhabitants of the homes of residents; therefore, there is a need for clear guidelines in the field of oral hygiene to support caregivers’ adequate healthcare. This study further showed that the oral health of the elderly revealed a poor situation in terms of dental status, periodontal situation, and cleanliness of the prosthesis, tongue, and oral cavity [12,13,14,15,16,17,18,19,20,21]. Impaired oral health is influenced by multiple general health problems, such as dementia, frailty, psychological disorders, malnutrition, and multiple-drug secondary therapy [10]. Poor oral health affects general health, and, in particular, nutritional status, cardiovascular disease, and aspiration pneumonia [22,23,24,25,26]. The oral health of older people living in LTCFs is compromised due to limitations on the part of caregivers: a lack of knowledge of dental hygiene; a lack of skills to deal with different types of prostheses; poor attitude towards, and low priority for, oral health; time limits; and high staff turnover [27,28].

However, interventions to improve this situation have been conducted through the educational training of nurses and/or caregivers [29,30]. The inclusion of a dental hygienist in the organization and education on oral health in the LTCFs appears to improve oral hygiene [31]. Moreover, frequent and regular support of tooth-brushing, denture cleaning, and professional dental care reduces the prevalence of aspiration pneumonia [32,33] and can improve malnutrition and poor appetite [32].

These measures highlight how in the case of oral health, as in other areas, support staff can improve the health of resident guests in long-term care and rest homes.

Furthermore, a pilot study conducted in Malaysia aimed at examining the association between the retirement home internal environment (RHIE), the basic requirements regulations (BRR) of nursing homes, and retirement home performance (RHP), as well as the elements considered critical to an optimal retirement home, gleaned from residents’ perceptions regarding ways to further improve nursing home services and operations in the community. In Malaysia, there are two types of retirement homes: ambient-assisted living concepts (AALCs), i.e., low- and middle-income retirement homes, which aim to reduce dependency on the caregiver and support independent living based on residential care, where new technologies and the social environment improve the quality of life of the elderly [33,34,35,36,37,38,39]; and long-term retirement home concepts (LTRHCs), which, unlike AALCs, face challenges in finding support, funding, donations, and sponsorships to support their operations. Most nongovernmental organizations or nonprofit organizations have to seek sponsorship or funding from the corporate sector to finance their day-to-day activities, treatments, and medicines. LTHRCs are set up to meet the basic needs of daily life, including shelter, food, and medicines, while the main goal of the AALCs is to use smart technology to enable older people to live independently for as long as possible [34,35,36,37,38,39].

This report, in relation to the indoor environment in nursing homes, focuses on the availability of skilled caregivers, and 24h professional and ambulance services, as well as an integrated database system in nursing homes. Healthcare professionals or nurses should be sufficiently qualified to ensure that residents receive the appropriate attention and services to ensure performance improvement. Disabled older adults require continuous attention and monitoring. Therefore, a well-trained carer or nurse needs to undergo specialized training to be prepared to provide help and support to tenants or occupiers and ensure that tenants acquire the best quality in terms of services [36]. Nursing homes should have a first aid kit and telephone line installed in every room to ensure immediate access to caregivers during emergencies and/or when accidents occur nearby. Furthermore, these houses must be equipped with an emergency button capable of alerting 24h ambulance services in case of need [37,39]. Moreover, it is a basic requirement for nursing home residents to have a good relationship with professional healthcare workers.

In this study, in line with the results of previous studies, we found that RHIE and BRR are critical elements for nursing homes; the most critical elements for RHIE were a good relationship between older adults and hospital professionals, older-centered treatment to keep older adults fit, active, and happy, an integrated database system containing tenant treatment information and medications, and 24h professional ambulance services. The BRRs related to nursing homes were the availability of transport and medical assistance, the multipurpose card and the provision of a food bank for the elderly, an integrated database accessible to the public at the nursing home, and efficient and effective space management in nursing homes; important elements for RHP included increasing the number of new check-ins and retained seniors, the quality of food and facilities, and screening equipment. Important elements for RHIE were older hospital professional relationships, treatment focused on elders, and 24h professional ambulance services [38,39]. Therefore, RHIE and BRR need to be streamlined to ensure that RHP aligns with the expectations of nursing home tenants and occupants [39].

Furthermore, in the international scientific literature, we can see how these guidelines and protocols can be implemented to optimize the organization, management, and personnel, and thus relieve health responsibility. An example is provided by the creation and implementation of an Infection Prevention and Control (IPAC) and a surgical wound analysis team SWAT team (IPAC-SWAT) activated by the health system as an innovative method in Canada during the initial phase of the SARS-CoV-2 pandemic in April 2020, when widespread outbreaks of COVID-19 occurred in long-term care facilities (LTCFs), retirement homes (RHs), and other care facilities around the world. This was a successful initiative that managed institutional outbreaks and promoted preparedness through staff education and training. Following IPAC-SWAT interventions, outbreaks were declared over an average of 20.6 days in facilities. IPAC-SWAT co-led the creation of written guidelines for outbreak management, adopted from best-practice literature and regional guidelines, which were distributed to partner houses to inform them of multidimensional interventions and supportive strategies to help manage and prepare for the COVID-19 outbreak through engagement, education, and training. This involved a strong focus on the sustainability of IPAC best practices through the education of IPAC champions [40].

In relation to health policy in Canada, in terms of the health management of publicly funded continuing care in Canada, retirement homes (RH) have been regulated since 2010 and are defined in legislation as a “residential development or part of a residential development”, “occupied primarily by persons aged 65 and over”, whose residents “are not related to the house manager” and have “at least two available care services, directly or indirectly”. RHs can provide a range of services to their residents and must include a minimum of two of the following required for a license: meals; assistance with bathing, grooming, dressing, or walking; administering medication; continence care; or making available a physician, nurse, or pharmacist. RHs are an option for older people who wish to move from a private home or apartment to an environment where services are available to meet present or anticipated needs or preferences and to live close to others with similar interests [41].

The needs of prospective residents are assessed prior to a tenancy agreement to ensure that adequate care is available (ORCA 2015). As a place of residence, RHs can be placed somewhere between private homes or apartments (PH) and nursing homes, called long-term care homes (LTCHs). The need for personal care or other healthcare services is not a requirement for moving to an RH, and some people who choose to relocate there are completely independent and self-sufficient. In addition, healthcare management includes long-term home healthcare in private homes and apartments and providing home healthcare services for various populations, including children and schools, short-term acute and rehabilitation, palliatives, and people who need support to stay in their own homes [41]. A 2017 study in Canada compared the care provided to home care patients with that provided to patients residing in long-term care facilities and showed that the latter had higher rates of dementia and moderate cognitive impairment. Home care systems were less supportive but had more personal care and nursing services than those provided by the public home care system, more frequent but shorter home support visits, and lower-than-expected public home care expenses. These lower costs may be due to efficient care delivery or nursing homes providing some services otherwise provided by the public home care system.

Dementia is also an essential feature of the elderly that needs to be considered in long-term facilities. Dementia and older age are often characterized by other psychological traits, such as anxiety, agitation, and apathy, which require psychological and psychiatric treatment. Isolation is an essential element that influences the outcome of such patients. Moreover, recent literature showed that the COVID-19 pandemic increased these issues by protracting isolation due to overlapping restrictions. Further improvement of the quality of life in elderly residencies in all countries is needed. In this regard, recent evidence suggests that dementia-specific residential facilities should be designed to compensate for disability, maximize independence, reinforce personal identity, enhance self-esteem/confidence, demonstrate care for staff, and welcome relatives and the local community. These standards of care need to be highlighted and related to the circumstances of the single case when dealing with a malpractice case regarding managing the elderly in long-term facilities in a forensic context [42,43,44,45].

Elderly people residing in nursing homes are at particular risk for physical, sexual, or psychological abuse and neglect. The prevalence of elderly abuse in institutions such as hospitals, nursing homes, and other long-term care facilities is estimated to be extremely high [46]. Most of the themes suffer from several chronic diseases that lead to physical and cognitive functioning limitations, and many cannot report abuse or neglect. Elder abuse is an important and widespread public health concern with social, health-related, and economic implications due to increased morbidity and premature mortality [47,48,49]. The phenomenon of elder abuse perpetrated by nurses is still unknown. Several studies show that care managers lack awareness of elder abuse and neglect [47,48,49,50,51,52,53]. The underestimated elderly abuse is mainly related to missing or poor reports from healthcare staff [50,51,52,53] and to the lack of available research in this field. It is mandatory to acquire necessary knowledge to identify the physical signs or suspected signs of abuse and neglect and specific training programs, as highlighted by some programs carried out in Italy and in some European countries, to improve the reporting procedures for prevention of abuse of the elderly in institutional settings [48,49,50,51,52,53].

The impact of the pandemic associated with coronavirus disease 2019 in the long-term care (LTC) services has been particularly acute related to the large numbers of older people dependent on care falling ill and because LTC workers are particularly exposed to the infection. As underlined by international epidemiological studies, the LTCF residents are at greater risk of COVID-19 and adverse consequences such as hospitalization and death [54,55,56,57,58,59]. The increasing trend of COVID-19-related mortality worldwide has led the political and health authorities to take urgent responses to contain and mitigate the pandemic diffusion in LTC, including the adoption of telemedicine in nursing homes [54,55,56,57,58,59]. Although people in all facilities are mostly older adults with high degrees of frailty and medical complexity, residents of long-term care homes show markedly higher needs [41]. It is also worth noting that telehealth technical opportunities in the era of the COVID-19 pandemic appear to have yielded results amenable to further implementation residences for elderly and sick people [60,61,62,63].

## 4. Conclusions

The presented cases offer a snapshot of the clinical and medico-legal issues of the assistance of the elderly in long-term care facilities. Residencies for elderly and sick people and self-sufficient and dependent persons are today highly varied and governed by multifaceted and complex regulatory dictates, but sometimes lacking for some categories of organizations and guests in relation to certain profiles of responsibility, including, in some cases, healthcare [63,64,65,66].

Additionally, in consideration of the current target of users who are assisted, increasingly characterized by a high degree of fragility and who sometimes require assistance not only in social and healthcare but also in the strict sense of health, interventions certainly appear appropriate for legislation, or at least the drafting/review of guidelines characterized by a certain degree of authority by the scientific societies of the sector that refer to the global assistance of the guest/patient and are aimed at filling a regulatory gap which, given the literature examined, currently affects several regions/states. This gap could create the possibility of encountering medico-legal problems that could compromise the patient as well as the structure, and, thus, filling the gap could improve the quality of the assistance provided.

To date, there is basic national legislation in various nations to which regulations are added with different characteristics from region to region and from structure to structure, as can be seen from the different types of contracts that each organization offers to its guests. Medical records accuracy needs to be vigorously promoted as it has a leading role in preventing medical malpractice liability and in the documentation of adherence to good clinical practices of care. Recommendations and evidence-based guidelines promotion, as well as surveillance of the maintenance of a high standard of care and the prevention of violence and abuse, should be implemented in the assistance of older adults in long-term care facilities, as older people’s safety represents a significant public health goal.

## Data Availability

Not applicable.
